# A pairwise pseudo-likelihood approach for regression analysis of left-truncated failure time data with various types of censoring

**DOI:** 10.1186/s12874-023-01903-x

**Published:** 2023-04-04

**Authors:** Li Shao, Hongxi Li, Shuwei Li, Jianguo Sun

**Affiliations:** 1 School of Economics and Statistics, Guangzhou University, Guangzhou, China; 2 Department of Statistics, University of Missouri, Columbia, Missouri, USA

**Keywords:** Cox model, EM algorithm, Interval censoring, Left truncation, Partly interval-censored data

## Abstract

**Background:**

Failure time data frequently occur in many medical studies and often accompany with various types of censoring. In some applications, left truncation may occur and can induce biased sampling, which makes the practical data analysis become more complicated. The existing analysis methods for left-truncated data have some limitations in that they either focus only on a special type of censored data or fail to flexibly utilize the distribution information of the truncation times for inference. Therefore, it is essential to develop a reliable and efficient method for the analysis of left-truncated failure time data with various types of censoring.

**Method:**

This paper concerns regression analysis of left-truncated failure time data with the proportional hazards model under various types of censoring mechanisms, including right censoring, interval censoring and a mixture of them. The proposed pairwise pseudo-likelihood estimation method is essentially built on a combination of the conditional likelihood and the pairwise likelihood that eliminates the nuisance truncation distribution function or avoids its estimation. To implement the presented method, a flexible EM algorithm is developed by utilizing the idea of self-consistent estimating equation. A main feature of the algorithm is that it involves closed-form estimators of the large-dimensional nuisance parameters and is thus computationally stable and reliable. In addition, an R package LTsurv is developed.

**Results:**

The numerical results obtained from extensive simulation studies suggest that the proposed pairwise pseudo-likelihood method performs reasonably well in practical situations and is obviously more efficient than the conditional likelihood approach as expected. The analysis results of the MHCPS data with the proposed pairwise pseudo-likelihood method indicate that males have significantly higher risk of losing active life than females. In contrast, the conditional likelihood method recognizes this effect as non-significant, which is because the conditional likelihood method often loses some estimation efficiency compared with the proposed method.

**Conclusions:**

The proposed method provides a general and helpful tool to conduct the Cox’s regression analysis of left-truncated failure time data under various types of censoring.

**Supplementary Information:**

The online version contains supplementary material available at 10.1186/s12874-023-01903-x.

## Introduction

Failure time data are frequently encountered in various scientific areas, including clinical trials, epidemiology surveys, and biomedical studies. A key feature of such data is the presence of censoring, which usually poses great computational challenges for their analysis [1, 2]. The type of censoring that has been investigated most is apparently right censoring [3–6]. Other types of censored data that often occur in practice include interval-censored and partly interval-censored data [7–13]. In particular, Gao et al. [10] recently proposed an efficient semiparametric estimation approach for the analysis of partly interval-censored data under the accelerated failure time model. Zhou et al. [13] also studied the analysis of partly interval-censored failure time but via the transformation models.

For failure time data, in addition to censoring, left truncation also often arises due to the use of cross-sectional sampling strategy and can substantially complicate the data analysis. For example, in the Canadian Study of Health and Aging Study, the failure time of interest is defined as the duration from the onset of dementia to death [14]. Since only dementia patients who had not experienced the death at the enrollment are included in the study, the patient’s death time is expected to suffer from left truncation, where the truncation time is the gap time between the onset of dementia and the enrollment. Therefore, the sampled patients are no longer representative of the whole population under study, and it is well-known that ignoring the left truncation in the data analysis often leads to biased parameter estimation.

Due to the ubiquity of left truncation in failure time studies, extensive efforts have been devoted to the method developments for the analysis of the left-truncated failure time data under various types of censoring scheme [15–25]. For instance, Wang et al. [16] considered the left-truncated and right-censored data, and developed a conditional estimation approach under the proportional hazards (PH) model, while Pan and Chappell [17] investigated the analysis of left-truncated and interval-censored data and suggested a marginal likelihood approach and a monotone maximum likelihood approach for the PH model. Gao and Chan [24] discussed the same model and data structure as Pan and Chappell [17], but further assumed that the truncation times follow the uniform distribution, which is usually referred to as the stationary or length-biased assumption in the literature. However, it is worth noting that this approach may produce biased parameter estimation when the length-biased assumption is violated in practical applications. For the left-truncated and partly interval-censored data, Wu et al. [25] provided a conditional likelihood approach for the PH model in the presence of a cured subgroup.

In addition to the work described above, Huang and Qin [14] also studied left-truncated and right-censored data and proposed an estimation procedure for the additive hazards model by combining a pairwise pseudo-score function and the conditional estimating function. This approach is appealing since it utilizes the marginal likelihood of the truncation times and can thus improve the estimation efficiency. In addition, the employed pairwise pseudo-likelihood can eliminate nuisance parameters from the marginal likelihood of the truncation times, leading to an estimating equation function with tractable form, and can yield more efficient estimation compared with the conditional estimating equation approach. Inspired by the work of Huang and Qin [14], Wu et al. [26] proposed a pairwise likelihood augmented estimator for the PH model with the left-truncated and right-censored data. Furthermore, Wang et al. [27] considered the analysis of left-truncated and interval-censored data with the PH model, and developed a sieve maximum likelihood estimation procedure by accommodating the pairwise likelihood function of the truncation times.

In the following, we will consider regression analysis of left-truncated failure time data under the PH model and various types of censoring mechanism, including the interval censoring, right censoring and a mixture of them. Specifically, motivated by Huang and Qin [14] and Wu et al. [26], we propose a nonparametric maximum likelihood estimation (NPMLE) approach by combining the conditional likelihood of the failure times with the pairwise likelihood obtained from the marginal likelihood of the truncation times, rendering an efficient estimation for the PH model. A flexible EM algorithm that can accommodate various types of censored data will be developed to implement the NPMLE. Through the desirable data augmentation, the objective function in the M-step of the algorithm has a tractable form, and one can estimate the regression coefficients and the nuisance parameters related to the cumulative baseline hazard function separately. In particular, by utilizing the spirit of self-consistent estimation equation, we obtain the explicit estimators of the possibly large-dimensional nuisance parameters, which can greatly relieve the computational burden in the optimization procedure. The numerical results obtained from extensive simulation studies demonstrate that the proposed method is computationally stable and reliable and can improve the estimation efficiency of the conditional likelihood approach. In other words, the proposed method provides a general and helpful tool to conduct the Cox’s regression analysis of left-truncated failure time data under various types of censoring.

The remainder of this paper is organized as follows. In Section Notation, model, and likelihood, we will first introduce some notation, data structure and the model, and then present the observed data likelihood function. Section Estimation procedure presents the developed EM algorithm to implement the NPMLE. In Section Simulation studies, extensive simulation studies are conducted to evaluate the empirical performance of the proposed method, followed by an application to a set of real data in Section An application. Section Discussion and concluding remarks gives some discussion and concluding remarks.

## Notation, model, and likelihood

Consider a failure time study involving left truncation, and for a subject from the target population, let $T^{*}$ denote the underlying failure time, that is, the time to the onset of the failure event. Let $A^{*}$ be the underlying truncation time (i.e. the time to the study enrolment), which is assumed to be independent of $T^{*}$, and $\varvec{Z}^{*}$ be the *p*-dimensional vector of covariates. For a subject enrolled in the study (i.e. satisfying $T^{*}\ge A^{*}$), denoted by *T*, *A* and $\varvec{Z}$ the failure time, the truncation time and the vector of covariates, respectively. Then $(T, A,\varvec{Z})$ has the same joint distribution as $(T^{*}, A^{*},\varvec{Z}^{*})$ conditional on $T^{*}\ge A^{*}$.

Let *f* and *S* denote the density and survival functions of $T^{*}$, respectively. Let *h* be the density function of $A^{*}$. Then the joint density function of (*T*, *A*) at (*t*, *a*) is

$$\begin{aligned} \frac{f(t)h(a)}{\int _0^{\infty }S(u)h(u)du}=\frac{f(t)}{S(a)}\times \frac{S(a)h(a)}{\int _0^{\infty }S(u)h(u)du}, \quad (0\le a\le t), \end{aligned}$$

where *f*(*t*)/*S*(*a*) is the conditional density of *T* given *A*, $S(a)h(a)/\int _0^{\infty }S(u)h(u)du$ is the marginal density of *A*. To describe the effect of $\varvec{Z}^{*}$ on the failure time $T^{*}$, we assume that $T^{*}$ follows the PH model with the conditional cumulative hazard function of $T^{*}$ given $\varvec{Z}^{*}$ taking the form

1 $$\begin{aligned} \Lambda (t \mid \varvec{Z}^{*})=\Lambda (t)\exp (\varvec{Z}^{*\top } \varvec{\beta }). \end{aligned}$$

In the above, $\Lambda (t)$ is an unspecified baseline cumulative hazard function and $\varvec{\beta }$ denotes a *p*-dimensional vector of regression coefficients.

As mentioned above, censoring always exists in failure time studies. Define $\Delta =1$ if *T* can be observed exactly and 0 otherwise. If $\Delta =0$, let (*L*, *R*] be the smallest interval that brackets *T* with $L \ge A$. Clearly, *T* is left-censored if $L = A$, *T* is right-censored if $R = \infty$, and *T* is interval-censored if $R < \infty$. In the sequel, notations with the subscript $_i$ represent the corresponding sample analogues. Therefore, we have partly interval-censored data if the obtained data consist of *n* independent observations denoted by $(A_i, T_i, \Delta _i, \varvec{Z}_i)$ if $\Delta _i=1$ and $(A_i, L_i, R_i, \Delta _i, \varvec{Z}_i)$ if $\Delta _i=0$ for $i = 1, \ldots , n$. Notably, the data above reduce to interval-censored data if $\Delta _i = 0$ for $i = 1, \ldots , n$, and right-censored data if $R_i = \infty$ for $i = 1, \ldots , n$.

Let $S(t \mid \varvec{Z}_i) = \exp \{-\Lambda (t)\exp (\varvec{Z}_i^{\top } \varvec{\beta })\}$ and $\lambda (t)= d\Lambda (t)/dt$. Assume that $(L_i, R_i)$ is conditionally independent of $(A^{*}, T^{*})$ given $A^{*} \le T^{*}$ and $\varvec{Z}^{*}$, and that $A^{*}$ is independent of $\varvec{Z}^{*}$, the observed data likelihood function takes the form

2 $$\begin{aligned} {L}_{n}(\varvec{\beta },\Lambda ,h)= L^{C}_{n}(\varvec{\beta },\Lambda ) \times L^{M}_{n}(\varvec{\beta },\Lambda ,h), \end{aligned}$$

where

$$\begin{aligned} {L}^{C}_{n}(\varvec{\beta },\Lambda ) = \prod _{i=1}^n{} & {} \frac{\{\lambda (t)\exp (\varvec{Z}_i^{\top } \varvec{\beta })S(T_i \mid \varvec{Z}_i)\}^{\Delta _i}\{S(L_i \mid \varvec{Z}_i)-S(R_i \mid \varvec{Z}_i)\}^{1-\Delta _i}}{S(A_i \mid \varvec{Z}_i)} \\ = \prod _{i=1}^n{} & {} \left[ \lambda (t)\exp (\varvec{Z}_i^{\top } \varvec{\beta })\exp \{-(\Lambda (T_i)-\Lambda (A_i))\exp (\varvec{Z}_i^{\top } \varvec{\beta })\}\right] ^{\Delta _i}\\{} & {} \times \left[ \exp \{-(\Lambda (L_i)-\Lambda (A_i))\exp (\varvec{Z}_i^{\top } \varvec{\beta })\} \right. \\{} & {} \left. -\exp \{-(\Lambda (R_i)-\Lambda (A_i))\exp (\varvec{Z}_i^{\top } \varvec{\beta })\}\right] ^{1-\Delta _i}, \end{aligned}$$

and

$$\begin{aligned} {L}^{M}_{n}(\varvec{\beta },\Lambda ,h) = \prod _{i=1}^{n}\frac{S(A_i \mid \varvec{Z}_i) h(A_i)}{\int _0^{\infty }S(u \mid \varvec{Z}_i)h(u)du}. \end{aligned}$$

In the above, $L^C_n(\varvec{\beta },\Lambda )$ is the conditional likelihood of $\{\Delta _i T_i, (1-\Delta _i)L_i, (1-\Delta _i)R_i, \Delta _i\}$ given $(A_i, \varvec{Z}_i)$, and $L^M_n(\varvec{\beta },\Lambda , h)$ is the marginal likelihood of $A_i$ given $\varvec{Z}_i$. Note that the observed data likelihood $L_n(\varvec{\beta },\Lambda ,h)$ has an intractable form due to the complex data structure and the involvement of the nuisance functions $\Lambda$ and *h*. For the estimation, it is apparent that performing direct maximization of $L_n(\varvec{\beta },\Lambda ,h)$ with respect to all parameters is quite challenging and unstable even after approximating $\Lambda$ and *h* with some smooth functions with finite-dimensional parameters. To address this issue, in the next section, we will develop a flexible EM algorithm by introducing some Poisson latent variables in the data augmentation procedure, which can greatly simplify the form of $L^C_n(\varvec{\beta },\Lambda )$. In addition, by following Liang and Qin [28] and others, we will employ the pairwise likelihood approach to eliminate the nuisance function *h* from the marginal likelihood $L^M_n(\varvec{\beta },\Lambda ,h)$. The above two manipulations make the estimation procedure appealing and easily implemented.

## Estimation procedure

To estimate $\varvec{\beta }$ and $\Lambda$, we adopt the NPMLE approach and develop an EM algorithm for its implementation. For this, we will first discuss the data augmentation and then present the pairwise likelihood method as well as the E-step and M-step of the algorithm.

### Data augmentation

First note that the likelihood function above depends on $\Lambda (t)$ only through its values at the finite observation times, exactly-observed failure times and truncation times. Let $t_1\cdots<t_{K_n}<\infty$ denote the ordered sequence of these unique time points, and assume that $\Lambda (t)$ is a step function at $t_k$ with the non-negative jump size $\lambda _k$ for $k = 1,\ldots , K_n$. Then the conditional likelihood $L^C_n(\varvec{\beta },\Lambda )$ can be re-expressed as

$$\begin{aligned} L^{C}_{1n}(\varvec{\theta })= \prod _{i=1}^n{} & {} \left[ \prod _{k=1}^{K_n}\lambda _k^{I(T_i=t_k)} \exp (\varvec{Z}_i^{\top } \varvec{\beta })\exp \left\{ -\sum _{A_i \le t_k\le T_i}\lambda _k\exp (\varvec{Z}_i^{\top } \varvec{\beta })\right\} \right] ^{\Delta _i} \\{} & {} \times \left[ \exp \left\{ -\sum _{A_i\le t_k\le L_i}\lambda _k\exp (\varvec{Z}_i^{\top } \varvec{\beta })\right\} \right. \\{} & {} \left. -I(R_i<\infty )\exp \left\{ -\sum _{A_i \le t_k\le R_i}\lambda _k\exp (\varvec{Z}_i^{\top } \varvec{\beta })\right\} \right] ^{1-\Delta _i}, \end{aligned}$$

where $$\varvec{\theta } = (\varvec{\beta }^{\top }, \lambda _1, \ldots , \lambda _{K_n})^{\top }$$.

To simplify $L^C_{1n}(\varvec{\theta })$, for the *i*th subject, we introduce a set of new independent latent variables $\{W_{ik}; k=1,2,\cdots ,K_n\}$ relating to $t_1, t_2,\cdots , t_{K_n}$ respectively, where $W_{ik}$ is a Poisson random variable with mean $\lambda _k\exp (\varvec{Z}_i^{\top } \varvec{\beta })$. Then $L^C_{1n}(\varvec{\theta })$ can be equivalently expressed as

$$\begin{aligned} L^C_{2n}{} & {} (\varvec{\theta })=\prod _{i=1}^n \left[ P\left( \sum _{A_i\le t_k< T_i}W_{ik}=0\right) P\left( W_{ik}|_{t_k = T_i}=1\right) \right] ^{\Delta _i}\\{} & {} \times \left[ P\left( \sum _{A_i\le t_k\le L_i}W_{ik}=0\right) P\left( \sum _{L_i< t_k\le R_i}W_{ik} > 0\right) ^{ I(R_i < \infty )} \right] ^{1-\Delta _i}, \end{aligned}$$

where $W_{ik}|_{t_k = T_i}$ denotes the variable in $\{W_{ik}; k=1,2,\cdots ,K_n\}$ that satisfies $t_k = T_i$.

Define $R_i^{*} = (1-\Delta _i) (L_i I(R_i=\infty )+ R_i I(R_i<\infty )) + \Delta _i T_i$, and let $p\{W_{ik} \mid \lambda _k\exp (\varvec{Z}_i^{\top } \varvec{\beta })\}$ be the probability mass function of $W_{ik}$ with mean $\lambda _k\exp (\varvec{Z}_i^{\top } \varvec{\beta })$. By treating the latent variables $W_{ik}$’s as observable, the augmented likelihood function is given by

$$\begin{aligned} L^C(\varvec{\theta })= & {} \prod _{i=1}^n \prod _{k=1}^{K_n} p\{W_{ik} \mid \lambda _k\exp (\varvec{Z}_i^{\top } \varvec{\beta })\}^{I(A_i \le t_k\le R_i^{*})}\\= & {} \prod _{i=1}^n \prod _{k=1}^{K_n} \left[ \frac{\{\lambda _k\exp (\varvec{Z}_i^{\top } \varvec{\beta })\}^{W_{ik}}}{W_{ik}!}\exp \{-\lambda _k\exp (\varvec{Z}_i^{\top } \varvec{\beta })\}\right] ^{I(A_i \le t_k\le R_i^{*})}, \end{aligned}$$

which subjects to the constraints that $\sum _{A_i\le t_k < T_i}W_{ik}=0$ and $W_{ik}|_{T_i = t_k}=1$ if $\Delta _i = 1$, $\sum _{A_i\le t_k\le L_i}W_{ik}=0$ and $\sum _{L_i< t_k\le R_i}W_{ik}>0$ if $\Delta _i = 0$ and $R_i < \infty$; and $\sum _{A_i\le t_k\le L_i}W_{ik}=0$ if $\Delta _i = 0$ and $R_i = \infty$.

### Pairwise likelihood

Since the density function *h* in the marginal likelihood $L^M_n(\varvec{\beta },\Lambda , h)$ is a nuisance function, we follow the work of Liang and Qin [28] and apply the pairwise likelihood method to $L^M_n(\varvec{\beta },\Lambda ,h)$ to eliminate *h*. Note that, for $i \ne j$, by conditioning on $(\varvec{Z}_i, \varvec{Z}_j)$ and having observed $(A_i, A_j)$ but without knowing the order of $A_i$ and $A_j$, the pairwise pseudo-likelihood of the observed $(A_i, A_j)$ is given by

$$\begin{aligned}{} & {} \frac{ \frac{S(A_i \, \mid \, \varvec{Z}_i)h(A_i)}{\int _0^{\infty }S(a \, \mid \, \varvec{Z}_i)h(a)da} \times \frac{S(A_j \, \mid \, \varvec{Z}_j)h(A_j)}{\int _0^{\infty }S(a \, \mid \, \varvec{Z}_j)h(a)da} }{ \frac{S(A_i \, \mid \, \varvec{Z}_i)h(A_i)}{\int _0^{\infty }S(a \, \mid \, \varvec{Z}_i)h(a)da} \times \frac{S(A_j \, \mid \, \varvec{Z}_j)h(A_j)}{\int _0^{\infty }S(a \, \mid \, \varvec{Z}_j)h(a)da} + \frac{S(A_i \, \mid \, \varvec{Z}_j)h(A_i)}{\int _0^{\infty }S(a \, \mid \, \varvec{Z}_j)h(a)da} \times \frac{S(A_j \, \mid \, \varvec{Z}_i)h(A_j)}{\int _0^{\infty }S(a \, \mid \, \varvec{Z}_i)h(a)da} }\\{} & {} =\frac{1}{1+R_{ij}(\varvec{\theta }) }, \end{aligned}$$

where

$$\begin{aligned} R_{ij}(\varvec{\theta })= & {} \frac{S(A_i \mid \varvec{Z}_j)S(A_j \mid \varvec{Z}_i)}{S(A_i \mid \varvec{Z}_i)S(A_j \mid \varvec{Z}_j)}\\= & {} \exp \left[ \sum _{k=1}^{K_n} \left\{ I(t_k\le A_i)-I(t_k\le A_j)\right\} \lambda _k \left\{ \exp (\varvec{Z}_i^{\top } \varvec{\beta })-\exp (\varvec{Z}_j^{\top } \varvec{\beta })\right\} \right] . \end{aligned}$$

Therefore, the pairwise likelihood $L^P_n(\varvec{\theta })$ of all pairs is given by

$$\begin{aligned} L^P (\varvec{\theta })=\prod _{i \ne j}\{1+R_{ij}(\varvec{\theta })\}^{-1}. \end{aligned}$$

Notably, through the above manipulation, $L^P (\varvec{\theta })$ depends on the parameters in the survival model, $\varvec{\beta }$ and $\lambda _1, \ldots , \lambda _{K_n}$, but not on the density function *h* of truncation time $A^{*}$.

### EM algorithm

Combing the augmented likelihood $L^C(\varvec{\theta })$ with the pairwise likelihood $L^P (\varvec{\theta })$, and taking into account the different magnitudes of $L^C(\varvec{\theta })$ and $L^P (\varvec{\theta })$, we can derive the composite complete-data log-likelihood as follows

$$\begin{aligned} l (\varvec{\theta }) = \frac{1}{n}\sum _{i=1}^n\sum _{k=1}^{K_n}{} & {} I(A_i\le t_k\le R_i^{*}) \left[ W_{ik}\log \{\lambda _k \exp (\varvec{Z}_i^{\top } \varvec{\beta }) \}-\lambda _k \exp (\varvec{Z}_i^{\top } \varvec{\beta }) \right. \\{} & {} \left. -\log (W_{ik}!)\right] -\frac{1}{n(n-1)}\sum _{i\ne j}\log \{1+R_{ij}(\varvec{\theta }) \}. \end{aligned}$$

In the E-step of the algorithm, we take the conditional expectations with respect to the latent variables $W_{ik}$’s in $l (\varvec{\theta })$, and for notational simplicity, we will ignore the conditional arguments including the observed data and the estimate of $\varvec{\theta }$ at the *l*th iteration denoted by $\varvec{\theta }^{(l)}$ in all conditional expectations. This step yields the following objective function

$$\begin{aligned} l_E(\varvec{\theta })= & {} \frac{1}{n}\sum _{i=1}^n\sum _{k=1}^{K_n}I(A_i\le t_k\le R_i^{*}) [E(W_{ik})\log \{\lambda _k\exp (\varvec{Z}_i^{\top } \varvec{\beta })\}-\lambda _k\exp (\varvec{Z}_i^{\top } \varvec{\beta })]\\{} & {} -\frac{1}{n(n-1)}\sum _{i\ne j}\log \{1+R_{ij}(\varvec{\theta }) \}. \end{aligned}$$

We now present the expressions of $E(W_{ik})$’s in $l_E(\varvec{\theta })$. Specifically, in the case of $\Delta _i = 1$ (exactly-observed $T_i$), we have $E(W_{ik})=0$ if $A_i\le t_k < T_i$, and $E(W_{ik})=1$ if $T_i=t_k$. In the case of $\Delta _i = 0$ and $A_i\le T_i\le L_i$ (left censoring), we have

$$\begin{aligned} E(W_{ik})=\frac{\lambda _k^{(l)}\exp (\varvec{Z}_i^{\top } \varvec{\beta }^{(l)})}{1-\exp \Big \{-\sum _{A_i \le t_k \le L_i}\lambda _k^{(l)}\exp (\varvec{Z}_i^{\top } \varvec{\beta }^{(l)}) \Big \} }, ~~~\textrm{if}~~~ A_i\le t_k\le L_i. \end{aligned}$$

In the case of $\Delta _i = 0$ and $R_i<\infty$ (interval censoring), we have $E(W_{ik})=0$ if $A_i\le t_k\le L_i$, and

$$\begin{aligned} E(W_{ik})=\frac{\lambda _k^{(l)}\exp (\varvec{Z}_i^{\top } \varvec{\beta }^{(l)})}{1-\exp \Big \{-\sum _{L_i<t_k \le R_i}\lambda _k^{(l)}\exp (\varvec{Z}_i^{\top } \varvec{\beta }^{(l)}) \Big \} }, ~~~\textrm{if}~~~ L_i< t_k\le R_i. \end{aligned}$$

In the case of $\Delta _i = 0$ and $R_i=\infty$ (right censoring), we have $E(W_{ik})=0$ if $A_i\le t_k\le L_i$.

Differentiating $l_E(\varvec{\theta })$ with respect to $\varvec{\beta }$ and $\lambda _k$’s yields the following composite score functions

$$\begin{aligned} U_{\varvec{\beta }}(\varvec{\theta })={} & {} \frac{1}{n}\sum _{i=1}^n\sum _{k=1}^{K_n}I(A_i\le t_k\le R_i^{*}) \varvec{Z}_i \{E(W_{ik})-\lambda _k\exp (\varvec{Z}_i^{\top } \varvec{\beta })\} \\ \nonumber{} & {} -\frac{1}{n(n-1)}\sum _{i\ne j}\frac{ \sum _{k=1}^{K_n} \lambda _k Q_{ij}^{(1)}(t_k)}{1+R_{ij}^{-1}(\varvec{\theta })}, \end{aligned}$$

and

$$\begin{aligned} U_{\lambda _k}(\varvec{\theta })={} & {} \frac{1}{n}\sum _{i=1}^nI(A_i\le t_k\le R_i^{*}) \left\{ \frac{E(W_{ik})}{\lambda _k} -\exp (\varvec{Z}_i^{\top } \varvec{\beta })\right\} \\{} & {} -\frac{1}{n(n-1)}\sum _{i\ne j} \frac{ Q_{ij}^{(0)}(t_k)}{1+R_{ij}^{-1}(\varvec{\theta })}, \end{aligned}$$

where $Q_{ij}^{(m)}(t;\varvec{\beta })=\Big \{\varvec{Z}_i^{\otimes m}\exp (\varvec{Z}_i^{\top } \varvec{\beta }) - \varvec{Z}_j^{\otimes m}\exp (\varvec{Z}_j^{\top } \varvec{\beta }) \Big \}\Big \{I(t\le A_i) -I(t\le A_j)\Big \}$ for $m=0$ or 1, $\varvec{Z}^{\otimes 0}=1$ and $\varvec{Z}^{\otimes 1}=\varvec{Z}$.

Specifically, at the $(l+1)$th iteration, based on estimating equation $U_{\lambda _k}(\varvec{\theta })=0$, one can derive a self-consistent solution to update each $\lambda _k$ :

3 $$\begin{aligned} \lambda _k^{(l+1)}=&\frac{\frac{1}{n}\sum _{i=1}^nI(A_i\le t_k\le R_i^{*})E(W_{ik}) }{\frac{1}{n}\sum _{i=1}^nI(A_i\le t_k\le R_i^{*})\exp (\varvec{Z}_i^{\top }\varvec{\beta }^{(l)}) + \frac{1}{n(n-1)}\sum _{i\ne j}\frac{Q_{ij}^{(0)}(t_k;\varvec{\beta }^{(l)})}{1+1/R_{ij}(\varvec{\theta }^{(l)}) } }. \end{aligned}$$

By combining the discussion above, the proposed EM algorithm can be summarized as follows:

Step 0: Choose initial values for $\varvec{\beta }^{(0)}$ and $\lambda _k^{(0)}$ for $k = 1, \ldots , K_n$, and set $l=0$.
Step 1: At the $(l+1)$th iteration, calculate each $E(W_{ik})$ based on the observed data and the parameter estimates at the *l*th iteration.
Step 2: Update each $\lambda _k$ with the closed-form expression (3).
Step 3: Update $\varvec{\beta }$ by solving the estimation equation $U_{\varvec{\beta }}(\varvec{\theta })=0$ with the one-step Newton-Raphson method, and increase *l* by 1.
Step 4: Repeat Steps 1 - 3 until the convergence is achieved.

The resulting estimators of $\varvec{\beta }$ and $\Lambda (t)$ are denoted as $\hat{\varvec{\beta }}$ and $\hat{\Lambda }(t)=\sum _{t_k\le t}\hat{\lambda }_k$, respectively, where $\hat{\lambda }_k$ is the estimate of $\lambda$ for $k = 1, \ldots , K_n$. For the standard error estimation of $\hat{\varvec{\beta }}$ and $\hat{\Lambda }(t)$, we propose to simply employ the nonparametric bootstrap approach ([29], for example), and the numerical results below suggest that it seems to work well in finite samples. The numerical results also indicate that the performance of the proposed algorithm is quite robust to the choices of the initial values of $\varvec{\beta }$ and $\lambda _k$’s. In the practical implementation of the proposed algorithm, one can simply set the initial value of each regression parameter to 0 and the initial value of each $\lambda _k$ to $1/K_n$. The algorithm is declared to achieve convergence if the sum of the absolute differences between two successive estimates of all parameters is less than a small positive constant, say 0.001. We implement the proposed algorithm under the Rcpp environment, which guarantees that the computation is efficient and tractable.

## Simulation studies

Simulation studies were conducted to assess the empirical performance of the proposed estimation procedure. In the study, the failure time $T^{*}$ was generated from model (1) with $\varvec{Z} = (Z_1, Z_2)^{\top }$, $Z_1\sim Bernoulli(0.5)$, $Z_2\sim Uniform(-0.5, 0.5)$, $\varvec{\beta } = (\beta _1, \beta _2)^{\top } = (1,1)^{\top },$ and $\Lambda (t)=t^2$, which corresponds to the Weibull distribution with the scale parameter 1 and the shape parameter 2. The truncation time $A^{*}$ was generated from either Uniform(0, $\tau ^{*}$) or exponential distribution with rate $\theta ^{*}$, where $\tau ^{*}$ or $\theta ^{*}$ was chosen to yield about $50\%$ average truncation rate. Note that when the truncation time follows the uniform distribution or satisfies the stationary assumption, we have the length-biased data, a special type of the left-truncated data as discussed above. Under the left truncation mechanism, the observed failure time *T* was equal to $T^{*}$ if $T^{*} > A^{*}$. We firstly considered the situation with left-truncated and partly interval-censored data. To construct censoring, for each subject, we mimicked the periodical follow-up study and generated a sequence of examination times with the first observation time being $A^{*}$ and the gap times of two successive observation times being $0.05+Uniform(0, 0.5)$. Then we used the above simulated failure time *T* instead of the interval-censored observation if interval length is less than 0.2 to construct the uncensored or exactly observed *T*. The length of study was set to be 1.5, beyond which no further examinations were conducted.

For comparison, we considered the following three competing methods: the proposed pairwise pseudo-likelihood method (Proposed method), the NPMLE method without adjusting for the left truncation (Ignoring truncation) and the conditional likelihood method (CL method). Specifically, in the supplementary materials, we developed an EM algorithm with Poisson latent variables to implement the conditional likelihood method, and the “Ignoring truncation” method can be implemented with the EM algorithm by setting each $A_i = 0$. We set $n = 100$, 300 or 500, and used 1000 replicates. Under the above configurations, the proportions of exactly-observed failure times ranged from $4\%$ to $26\%$; left censoring rates ranged from $16\%$ to $37\%$; right censoring rates ranged from $7\%$ to $33\%$ and interval censoring rates ranged from $24\%$ to $58\%$.

Table 1 presents the simulation results for the estimated regression parameters and the cumulative hazards function at $t=0.4$, 0.8 or 1.2 with partly interval-censored data. They include the estimated bias (Bias) given by the average of the 1000 estimates minus the true value, the sample standard error (SSE) of the 1000 estimates, the average of the 1000 standard error estimates (SEE), and the 95% empirical coverage probability (CP) yielded by the normal approximation. Specifically, the standard errors of the proposed pairwise pseudo-likelihood estimators were calculated via the nonparametric bootstrapping with 100 bootstrap samples. For CL and “Ignoring truncation” methods, we followed Zeng et al. [30] and proposed to adopt the profile likelihood approach to perform the variance estimation. This approach is simple and easy to implement, but can only provide the variance estimation for the estimated regression parameter, finite-dimensional parameter of interest. Thus, the SEEs of the cumulative hazards function estimates of the CL and “Ignoring truncation” methods were not available in Table 1. Given that $\Lambda (t)$ is always positive, we used the log-transformation and constructed its confidence band with the delta method as Mao and Lin [31] among others. For any *t*, the confidence interval of $\Lambda (t)$ is given by $[\hat{\Lambda }(t) \exp \{-z_{0.975}\hat{\sigma }(t)/\hat{\Lambda }(t)\},$ $\hat{\Lambda }(t) \exp \{z_{0.975}\hat{\sigma }(t)/\hat{\Lambda }(t)\}]$, where $\hat{\sigma }(t)$ is the standard error estimate of $\hat{\Lambda }(t)$, and $z_{0.975}$ is the upper 97.5th percentile of the standard normal distribution.

**Table 1:** Simulation results with partly interval-censored data, including the estimated bias (Bias), the sample standard error (SSE) of the estimates, the average of the standard error estimates (SEE), and the 95% empirical coverage probability (CP)

			Proposed method					CL method					Ignoring truncation			
*n*	Par	True	Bias	SSE	SEE	CP		Bias	SSE	SEE	CP		Bias	SSE	SEE	CP
$A^{*}$ follows the uniform distribution																
100	$\beta _1$	1	0.041	0.246	0.254	96.6		0.061	0.290	0.278	92.7		0.192	0.273	0.311	94.1
	$\beta _2$	1	0.045	0.403	0.408	95.6		0.055	0.492	0.467	93.7		0.180	0.474	0.445	90.8
	$\Lambda (0.4)$	0.16	0.009	0.094	0.087	95.6		0.002	0.092	**–**	**–**		-0.084	0.043	**–**	**–**
	$\Lambda (0.8)$	0.64	-0.037	0.165	0.161	93.9		-0.048	0.164	**–**	**–**		-0.245	0.103	**–**	**–**
	$\Lambda (1.2)$	1.44	-0.038	0.240	0.248	93.5		-0.067	0.240	**–**	**–**		-0.445	0.209	**–**	**–**
300	$\beta _1$	1	0.008	0.134	0.129	93.5		0.013	0.156	0.151	93.1		0.120	0.148	0.306	95.9
	$\beta _2$	1	0.012	0.212	0.212	94.6		0.025	0.248	0.253	95.2		0.146	0.244	0.305	92.4
	$\Lambda (0.4)$	0.16	0.020	0.067	0.064	94.2		0.020	0.067	**–**	**–**		-0.081	0.026	**–**	**–**
	$\Lambda (0.8)$	0.64	0.020	0.104	0.105	95.3		0.020	0.107	**–**	**–**		-0.240	0.061	**–**	**–**
	$\Lambda (1.2)$	1.44	-0.028	0.179	0.189	96.5		-0.024	0.182	**–**	**–**		-0.436	0.116	**–**	**–**
500	$\beta _1$	1	0.014	0.101	0.099	95.4		0.018	0.115	0.117	96.0		0.154	0.101	0.197	79.6
	$\beta _2$	1	0.014	0.161	0.163	94.6		0.020	0.191	0.193	95.0		0.146	0.193	0.219	85.7
	$\Lambda (0.4)$	0.16	0.012	0.048	0.048	96.7		0.012	0.048	**–**	**–**		-0.081	0.018	**–**	**–**
	$\Lambda (0.8)$	0.64	0.010	0.076	0.075	95.0		0.009	0.077	**–**	**–**		-0.244	0.044	**–**	**–**
	$\Lambda (1.2)$	1.44	-0.012	0.133	0.131	94.6		-0.012	0.135	**–**	**–**		-0.443	0.094	**–**	**–**
$A^{*}$ follows the exponential distribution																
100	$\beta _1$	1	0.045	0.242	0.251	94.9		0.062	0.272	0.266	93.1		0.146	0.266	0.294	95.3
	$\beta _2$	1	0.047	0.396	0.405	95.3		0.071	0.451	0.453	95.3		0.149	0.435	0.435	90.5
	$\Lambda (0.4)$	0.16	0.009	0.083	0.080	95.5		0.009	0.085	**–**	**–**		-0.068	0.046	**–**	**–**
	$\Lambda (0.8)$	0.64	-0.036	0.159	0.160	93.7		-0.038	0.159	**–**	**–**		-0.178	0.114	**–**	**–**
	$\Lambda (1.2)$	1.44	-0.042	0.234	0.240	92.7		-0.042	0.235	**–**	**–**		-0.280	0.251	**–**	**–**
300	$\beta _1$	1	0.011	0.131	0.133	95.9		0.016	0.147	0.148	94.9		0.084	0.137	0.250	97.4
	$\beta _2$	1	-0.001	0.210	0.217	95.9		0.007	0.228	0.246	96.7		0.082	0.229	0.294	95.6
	$\Lambda (0.4)$	0.16	0.017	0.053	0.053	96.5		0.017	0.054	**–**	**–**		-0.065	0.025	**–**	**–**
	$\Lambda (0.8)$	0.64	0.014	0.097	0.093	93.8		0.013	0.100	**–**	**–**		-0.173	0.066	**–**	**–**
	$\Lambda (1.2)$	1.44	-0.016	0.184	0.182	94.5		-0.015	0.184	**–**	**–**		-0.300	0.140	**–**	**–**
500	$\beta _1$	1	0.012	0.100	0.101	95.2		0.016	0.115	0.113	94.4		0.069	0.116	0.263	92.3
	$\beta _2$	1	0.010	0.165	0.167	94.7		0.008	0.187	0.188	94.7		0.097	0.173	0.247	92.3
	$\Lambda (0.4)$	0.16	0.014	0.044	0.045	95.3		0.015	0.044	**–**	**–**		-0.067	0.020	**–**	**–**
	$\Lambda (0.8)$	0.64	0.013	0.075	0.073	94.6		0.013	0.077	**–**	**–**		-0.174	0.061	**–**	**–**
	$\Lambda (1.2)$	1.44	-0.003	0.136	0.138	97.5		-0.003	0.138	**–**	**–**		-0.292	0.118	**–**	**–**

Note: “Proposed method” denotes the proposed pairwise pseudo-likelihood method, “CL method” denotes the conditional likelihood method, and “Ignoring truncation” denotes the NPMLE approach that ignores the existence of left truncation

One can see from Table 1 that the estimators of the proposed pairwise pseudo-likelihood method are virtually unbiased, the corresponding sample standard error estimates are close to the average standard error estimates, and the empirical coverage probabilities are all around the nominal value 95%, implying that the normal approximation of the asymptotic distribution of the proposed estimator seems reasonable. In addition, one can clearly find that the proposed method is more efficient than the conditional likelihood method, and this efficiency gain can be anticipated since the proposed method utilizes the information of the marginal distribution of the truncation time. Since the generated data are subject to biased sampling, as seen from Table 1, the “Ignoring truncation” method is expected to yield much larger estimation biases than the proposed and the conditional likelihood methods.

In the second study, we considered the left-truncated and interval-censored data. For this, we generated the truncation time $A^{*}$ in the same way as before, and set the first examination time being $A^{*}$. The gap time of two successive observation times was set to be $0.05+Uniform(0, 0.5)$, and the other model specifications were kept the same as above. Then we had the left-truncated and interval-censored data by contrasting the generated *T* with the observation times. Under the aforementioned simulation setups, the left censoring rates were from $20\%$ to $56\%$; the right censoring rates ranged from $7\%$ to $32\%$; interval censoring rates ranged from $27\%$ to $67\%$. The simulation results summarized in Table 2 again indicate that the proposed method performs reasonably well and has some advantages over the conditional likelihood and the “Ignoring truncation” methods.

**Table 2:** Simulation results with interval-censored data, including the estimated bias (Bias), the sample standard error (SSE) of the estimates, the average of the standard error estimates (SEE), and the 95% empirical coverage probability (CP)

			Proposed method					CL method					Ignoring truncation			
*n*	Par	True	Bias	SSE	SEE	CP		Bias	SSE	SEE	CP		Bias	SSE	SEE	CP
$A^{*}$ follows the uniform distribution																
100	$\beta _1$	1	0.057	0.253	0.258	96.1		0.100	0.302	0.262	89.5		0.212	0.290	0.323	93.3
	$\beta _2$	1	0.079	0.408	0.411	95.2		0.114	0.510	0.427	87.4		0.214	0.471	0.451	88.2
	$\Lambda (0.4)$	0.16	0.013	0.103	0.104	94.0		0.009	0.103	**–**	**–**		-0.076	0.052	**–**	**–**
	$\Lambda (0.8)$	0.64	-0.053	0.186	0.184	93.9		-0.039	0.185	**–**	**–**		-0.236	0.113	**–**	**–**
	$\Lambda (1.2)$	1.44	-0.033	0.306	0.301	92.1		-0.065	0.308	**–**	**–**		-0.431	0.230	**–**	**–**
300	$\beta _1$	1	0.025	0.134	0.132	93.9		0.042	0.155	0.150	93.1		0.154	0.149	0.209	90.3
	$\beta _2$	1	0.016	0.212	0.214	95.3		0.032	0.249	0.244	93.2		0.169	0.250	0.240	83.4
	$\Lambda (0.4)$	0.16	0.023	0.075	0.073	96.5		0.022	0.076	**–**	**–**		-0.068	0.034	**–**	**–**
	$\Lambda (0.8)$	0.64	-0.006	0.131	0.132	94.2		0.002	0.127	**–**	**–**		-0.224	0.069	**–**	**–**
	$\Lambda (1.2)$	1.44	-0.021	0.229	0.222	95.2		-0.017	0.225	**–**	**–**		-0.427	0.139	**–**	**–**
500	$\beta _1$	1	0.005	0.100	0.100	96.1		0.017	0.121	0.115	94.3		0.132	0.107	0.189	86.6
	$\beta _2$	1	0.022	0.165	0.163	93.6		0.036	0.202	0.189	91.8		0.148	0.188	0.179	75.4
	$\Lambda (0.4)$	0.16	0.019	0.059	0.055	93.9		0.018	0.063	**–**	**–**		-0.066	0.028	**–**	**–**
	$\Lambda (0.8)$	0.64	-0.014	0.098	0.101	95.1		-0.008	0.097	**–**	**–**		-0.222	0.057	**–**	**–**
	$\Lambda (1.2)$	1.44	-0.022	0.186	0.182	94.6		-0.019	0.189	**–**	**–**		-0.429	0.107	**–**	**–**
$A^{*}$ follows the exponential distribution																
100	$\beta _1$	1	0.084	0.250	0.266	96.8		0.115	0.292	0.259	88.9		0.164	0.273	0.309	94.2
	$\beta _2$	1	0.084	0.411	0.428	96.3		0.125	0.484	0.424	88.9		0.162	0.449	0.437	90.9
	$\Lambda (0.4)$	0.16	0.008	0.096	0.101	96.2		0.007	0.097	**–**	**–**		-0.062	0.056	**–**	**–**
	$\Lambda (0.8)$	0.64	-0.046	0.178	0.174	93.6		-0.05	0.179	**–**	**–**		-0.169	0.134	**–**	**–**
	$\Lambda (1.2)$	1.44	-0.043	0.289	0.303	93.5		-0.037	0.290	**–**	**–**		-0.267	0.272	**–**	**–**
300	$\beta _1$	1	0.030	0.136	0.135	94.3		0.046	0.152	0.145	92.5		0.126	0.145	0.179	91.4
	$\beta _2$	1	0.019	0.223	0.221	94.9		0.034	0.254	0.239	93.0		0.136	0.248	0.218	82.1
	$\Lambda (0.4)$	0.16	0.021	0.066	0.068	95.9		0.023	0.067	**–**	**–**		-0.056	0.035	**–**	**–**
	$\Lambda (0.8)$	0.64	-0.012	0.117	0.116	94.4		-0.005	0.119	**–**	**–**		-0.169	0.079	**–**	**–**
	$\Lambda (1.2)$	1.44	-0.027	0.206	0.211	95.1		-0.235	0.205	**–**	**–**		-0.288	0.161	**–**	**–**
500	$\beta _1$	1	0.014	0.105	0.102	94.5		0.024	0.116	0.111	93.5		0.100	0.110	0.187	90.4
	$\beta _2$	1	0.017	0.168	0.168	95.0		0.027	0.191	0.185	94.2		0.103	0.190	0.185	82.7
	$\Lambda (0.4)$	0.16	0.019	0.054	0.052	97.7		0.017	0.054	**–**	**–**		-0.055	0.027	**–**	**–**
	$\Lambda (0.8)$	0.64	-0.006	0.096	0.099	96.5		-0.003	0.094	**–**	**–**		-0.169	0.063	**–**	**–**
	$\Lambda (1.2)$	1.44	-0.002	0.184	0.184	95.1		-0.200	0.186	**–**	**–**		-0.288	0.131	**–**	**–**

Note: “Proposed method” denotes the proposed pairwise pseudo-likelihood method, “CL method” denotes the conditional likelihood method, and “Ignoring truncation” denotes the NPMLE approach that ignores the existence of left truncation

Note that Wu et al. [26] considered the left-truncated and right-censored data and proposed an iterative estimation procedure to implement the pairwise pseudo-likelihood method. It is clear that the proposed method can deal with such data too. Therefore, one may be interested in comparing the performance of the proposed method with that of Wu et al. [26]. To investigate this, we generated the failure time $T^{*}$ from model (1) with $\varvec{Z} = (Z_1, Z_2)^{\top }$, $Z_1\sim Bernoulli(0.5)$, $Z_2\sim Uniform(-1,1)$, $\beta _1 = \beta _2 = 1$, and $\Lambda (t)=t^2$. The truncation time $A^{*}$ was generated in the same way as before. The right censoring time *C* was generated independently from $Uniform(0,C_{max})$, where $C_{max}$ were chosen to yield about $30\%$ right censoring rate. The results given in Table 3 imply that the two methods can both perform well and give similar performance.

**Table 3:** Simulation results for the comparison of the proposed method with Wu et al. (2018)’s method under right censored data, including the estimated bias (Bias), the sample standard error (SSE) of the estimates, the average of the standard error estimates (SEE), and the 95% empirical coverage probability (CP)

		Proposed method					Wu et al. (2018)’s method			
*n*	Par	True	Bias	SSE	SEE	CP	Bias	SSE	SEE	CP
$A^{*}$ follows the uniform distribution										
100	$\beta _1$	1	0.025	0.244	0.247	95.0	0.026	0.244	0.227	92.8
	$\beta _2$	1	0.027	0.391	0.398	94.9	0.027	0.391	0.368	93.6
300	$\beta _1$	1	0.011	0.129	0.133	96.0	0.012	0.129	0.130	95.2
	$\beta _2$	1	0.005	0.246	0.216	94.8	0.005	0.216	0.211	95.1
500	$\beta _1$	1	0.005	0.100	0.102	95.2	0.005	0.100	0.100	95.1
	$\beta _2$	1	0.002	0.166	0.165	95.1	0.003	0.166	0.162	94.9
$A^{*}$ follows the exponential distribution										
100	$\beta _1$	1	0.024	0.248	0.257	95.8	0.024	0.248	0.237	94.3
	$\beta _2$	1	0.015	0.398	0.416	95.7	0.015	0.398	0.383	93.6
300	$\beta _1$	1	0.003	0.134	0.138	95.6	0.003	0.134	0.135	95.3
	$\beta _2$	1	0.008	0.218	0.223	95.4	0.008	0.218	0.219	95.2
500	$\beta _1$	1	0.010	0.107	0.106	94.7	0.010	0.107	0.105	95.2
	$\beta _2$	1	0.011	0.172	0.171	94.8	0.011	0.172	0.169	94.6

## An application

We apply the proposed method to a set of real data arising from the Massachusetts Health Care Panel Study (MHCPS) discussed in Pan and Chappell [17], Gao and Chan [24] and others. In 1975, the MHCPS enrolled elderly people who had not lost the active life in Massachusetts to evaluate the effect of gender (male or female) on the time to loss of active life. To determine when individuals in the study lost the active life, three subsequent follow-ups were taken at the 1.25, 6, and 10 years after the study enrolment. Therefore, age of the loss of active life, the defined failure time of interest $T^{*}$, cannot be recorded exactly and suffered from interval censoring. In the MHCPS, since subjects who had lost the active life before the study were not enrolled, the age of the loss of active life was subject to left truncation with the truncation time $A^{*}$ being the age at enrolment [17]. Therefore, we had left-truncated and interval-censored data. After deleting a small amount of unrealistic records of the raw data, 1025 subjects with the age ranging from 65 to 97.3 were considered in the current analysis. In particular, the right censoring rate is $45.8\%$.

Define $Z =1$ if the individual is male and 0 otherwise. For the analysis of the MHCPS data, as in the simulation studies, we considered three competing methods: the proposed pairwise pseudo-likelihood method (Proposed method), the conditional likelihood approach (CL method), and the NPMLE method that ignores the existence of left truncation (Ignoring truncation). Table 4 presents the obtained results including the estimated covariate effect (Est), the standard error estimate (Std) and the associated *p*-value for testing the covariate effect being zero. In the proposed pairwise pseudo-likelihood method, as in the simulation study, we employed the nonparametric bootstrapping with 100 bootstrap samples to calculate the standard error of the estimated regression parameter.

**Table 4:** Analysis results of the MHCPS data, including the estimated covariate effect (Est), the standard error estimate (Std) and the *p*-value

Method	Est	Std	*p*-value
Proposed method	0.122	0.060	0.041
CL method	0.133	0.082	0.103
Ignoring truncation	0.156	0.095	0.100

Note: “Proposed method” denotes the proposed pairwise pseudo-likelihood method, “CL method” denotes the conditional likelihood method, and “Ignoring truncation” denotes the NPMLE approach that ignores the existence of left truncation

One can see from Table 4 that the estimated coefficient and the standard error estimate of the proposed method are given by 0.122 and 0.060, respectively, meaning that males have significantly higher risk of losing active life than females. This conclusion is in accordance with that given in Gao and Chan [24] where the length-biased assumption was made for the truncation time. One can also find from Table 4 that the CL method recognized the covariate effect as non-significant, which is different from the conclusion obtained by the proposed method. This phenomenon may arise partly due to the fact the CL method often loses some estimation efficiency compared with the proposed method. Moreover, the results given in Table 4 suggested that the NPMLE method that ignores the existence of left truncation tended to overestimate the covariate effect, and this effect was also recognized as non-significant.

## Discussion and concluding remarks

In the preceding sections, we proposed a general or unified pairwise pseudo-likelihood approach for the analysis of left-truncated failure time data under the PH model. The proposed method is quite general and flexible since it applies to various types of censored data, including the partly interval-censored, interval-censored, and right-censored data. We devised an EM algorithm to calculate the nonparametric maximum likelihood estimators, which was shown to be computationally stable and reliable in finite samples. Numerical results indicated that, by utilizing the pairwise order information of the truncation times, the proposed method can indeed yield more efficient estimators compared with the conventional conditional likelihood estimation approach. An application to the MHCPS data demonstrated the practical utility of the proposed method.

Notably, in the proposed algorithm, the derivation of the self-consistent solution (3) for $\lambda _k$ is the desirable feature, which avoids the use of high-dimensional optimization procedure. In addition, the estimation equation $U_{\varvec{\beta }}(\varvec{\theta })=0$ for $\varvec{\beta }$ has tractable form and can be readily solved with some routine optimization procedure, such as the Newton-Raphson method. The two desirable features both make the proposed algorithm computationally stable and reliable. There may also exist some shortcomings of the proposed method. One is that the self-consistent solution (3) may not ensure that the estimate of $\lambda _k$ is always non-negative. However, it has been our experience that, given a reasonable initial value, the negative estimate of $\lambda _k$ is unlikely to occur in the simulations. As an alternative, by following Zhou et al. [32] and others, one can attempt to reparameterize each $\lambda _k$ as $\exp (\lambda _k^{*})$, where $\lambda _k^{*}$ is the unconstrained parameter to be estimated. Another is that we adopted the nonparametric bootstrap method to calculate the variance of parameter estimate, which involves repeated data sampling. This procedure will become computationally intensive if the sample size is extremely large. Future efforts will be devoted to develop a simple variance estimation procedure.

There may also exist several potential research directions for future research. One is that in the proposed method, we made a non-informative or independent censoring assumption [33, 34]. In other words, the failure times of interest were assumed to be conditionally independent of the observation times given the covariates. However, it is apparent that this assumption may not hold in some applications, and thus the generalizing of the proposed method to the situation of informative censoring deserves further investigation. In some applications, one may also encounter bivariate or multivariate failure time data [35], and it would be helpful to generalize the proposed method to deal with such data. Also the extensions of the proposed method to other regression models such as the transformation or additive hazards models can be useful.

## Supplementary Information

**Additional file 1.**

## Data Availability

The MHCPS data set used in this study can be downloaded at https://onlinelibrary.wiley.com/action/downloadSupplement?doi=10.1111%2Fj.0006-341X.2002.00064.x &file=BIOM_64_sm_010423.txt. The proposed algorithm can be implemented in the R package LTsurv, which is publicly available at https://github.com/lishuwstat/Left-truncation-Cox-Pairwise-likelihood.

## Abbreviations

PH: Proportional hazards
NPMLE: Nonparametric maximum likelihood estimation
EM: Expectation Maximization Algorithm
CL: Conditional likelihood
SSE: Sample standard error
SEE: Standard error estimate
CP: Coverage probability
MHCPS: Massachusetts Health Care Panel Study
