# Supplementary material for: A pairwise pseudo-likelihood approach for regression analysis of left-truncated failure time data with various types of censoring
Source: BMC Med Res Methodol. 2023 Apr 4;23:82. doi: 10.1186/s12874-023-01903-x (PMC10071649; doi:10.1186/s12874-023-01903-x)
Supplement: Supplementary file 1 — Additional file 1. [file 12874_2023_1903_MOESM1_ESM.pdf]

Supplementary Materials for "A Pairwise Pseudo-Likelihood Approach for Regression Analysis of Left-Truncated Failure Time Data with Various Types of Censoring"

Li Shao[1], Hongxi Li[1], Shuwei Li[1], Jianguo Sun[2]

[1] School of Economics and Statistics, Guangzhou University, Guangzhou, China

[2] Department of Statistics, University of Missouri,Columbia, Missouri, USA

January 30, 2023

Appendix: An EM Algorithm for Maximizing the Conditional Likelihood

The observed data conditional likelihood is given by

$$
\begin{aligned}
L_n^C(\boldsymbol{\beta}, \Lambda) =& \prod_{i=1}^{n} \frac{\{\lambda(t)\exp(\boldsymbol{Z}_i^\top \boldsymbol{\beta}) S(T_i \mid \boldsymbol{Z}_i)\}^{\Delta_i} \{S(L_i \mid \boldsymbol{Z}_i) - S(R_i \mid \boldsymbol{Z}_i)\}^{1-\Delta_i}}{S(A_i \mid \boldsymbol{Z}_i)} \\
=& \prod_{i=1}^{n} \left[\lambda(t)\exp(\boldsymbol{Z}_i^\top \boldsymbol{\beta}) \exp\{-(\Lambda(T_i) - \Lambda(A_i))\exp(\boldsymbol{Z}_i^\top \boldsymbol{\beta})\}\right]^{\Delta_i} \\
& \times \left[\exp\{-(\Lambda(L_i) - \Lambda(A_i))\exp(\boldsymbol{Z}_i^\top \boldsymbol{\beta})\} - \exp\{-(\Lambda(R_i) - \Lambda(A_i))\exp(\boldsymbol{Z}_i^\top \boldsymbol{\beta})\}\right]^{1-\Delta_i}.
\end{aligned}
$$

We assume that $\Lambda(t)$ is a step function with finite jump sizes at $t_1 \cdots < t_{K_n}$, the ordered sequence of unique finite observation times, exactly-observed failure times and truncation times. The non-negative jump size of $\Lambda(t)$ at t_k is denoted as λ_k for $k = 1, \ldots, K_n$. Then $L_n^C(\boldsymbol{\beta}, \Lambda)$ can

be rewritten as

$$L_{1n}^{C}(\boldsymbol{\theta}) = \prod_{i=1}^{n}\left[\prod_{k=1}^{K_n}\lambda_k^{I(T_i=t_k)}\exp(\boldsymbol{Z}_i^\top\boldsymbol{\beta})\exp\left\{-\sum_{A_i\le t_k\le T_i}\lambda_k\exp(\boldsymbol{Z}_i^\top\boldsymbol{\beta})\right\}\right]^{\Delta_i}$$
$$\times\left[\exp\left\{-\sum_{A_i\le t_k\le L_i}\lambda_k\exp(\boldsymbol{Z}_i^\top\boldsymbol{\beta})\right\}-I(R_i<\infty)\exp\left\{-\sum_{A_i\le t_k\le R_i}\lambda_k\exp(\boldsymbol{Z}_i^\top\boldsymbol{\beta})\right\}\right]^{1-\Delta_i},$$

where $\boldsymbol{\theta}$ is a vector containing all parameters to be estimated.

For the ith subject, we introduce a set of new independent latent variables $\{W_{ik}; k=1,2,\cdots,K_n\}$, where W_{ik} is a Poisson random variable with mean $\lambda_k\exp(\boldsymbol{Z}_i^\top\boldsymbol{\beta})$. Then we find that $L_{1n}^{C}(\boldsymbol{\theta})$ can be equivalently expressed with Poisson variables as

$$L_{2n}^{C}(\boldsymbol{\theta}) = \left[P\left(\sum_{A_i\le t_k<T_i}W_{ik}=0\right)\prod_{k=1}^{K_n}P\left(W_{ik}=1\right)^{I(T_i=t_k)}\right]^{\Delta_i}$$
$$=\left[P\left(\sum_{A_i\le t_k\le L_i}W_{ik}=0\right)P\left(\sum_{L_i<t_k\le R_i}W_{ik}>0\right)^{I(R_i<\infty)}\right]^{1-\Delta_i}$$

Define $R_i^*=(1-\Delta_i)(L_iI(R_i=\infty)+R_iI(R_i<\infty))+\Delta_iT_i$. By treating the latent variables W_{ik}'s as observable, the complete data likelihood function is given by

$$L^C(\boldsymbol{\theta})=\prod_{i=1}^{n}\prod_{k=1}^{K_n}\left[\frac{\{\lambda_k\exp(\boldsymbol{Z}_i^T\boldsymbol{\beta})\}^{W_{ik}}}{W_{ik}!}\exp\{-\lambda_k\exp(\boldsymbol{Z}_i^\top\boldsymbol{\beta})\}\right]^{I(A_i\le t_k\le R_i^*)},$$

which subjects to the constraints that $\sum_{A_i\le t_k<T_i}W_{ik}=0$ and $W_{ik}|_{T_i=t_k}=1$ if $\Delta_i=1$, $\sum_{A_i\le t_k\le L_i}W_{ik}=0$ and $\sum_{L_i<t_k\le R_i}W_{ik}>0$ if $\Delta_i=0$ and $R_i<\infty$; and $\sum_{A_i\le t_k\le L_i}W_{ik}=0$ if $\Delta_i=0$ and $R_i=\infty$.

In the E-step, we take conditional expectations with respect to all latent variables in $\log\{L^C(\boldsymbol{\theta})\}$, which yields

$$Q(\boldsymbol{\theta})=\frac{1}{n}\sum_{i=1}^{n}\sum_{k=1}^{K_n}I(A_i\le t_k\le R_i^*)[E(W_{ik})\log\{\lambda_k\exp(\boldsymbol{Z}_i^\top\boldsymbol{\beta})\}-\lambda_k\exp(\boldsymbol{Z}_i^\top\boldsymbol{\beta})].$$

We ignored the conditional arguments including the observed data and the estimate of $\boldsymbol{\theta}$ at the lth iteration denoted by $\boldsymbol{\theta}^{(l)}$ in the the expressions of $E(W_{ik})$'s, which have the form: $E(W_{ik})=0$ if $\Delta_i=1$ and $A_i\le t_k<T_i$, and $E(W_{ik})=1$ if $\Delta_i=1$ and $T_i=t_k$; $E(W_{ik})=0$ if $\Delta_i=0$ and $A_i\le t_k\le L_i$, and

$$E(W_{ik})=\frac{\lambda_k\exp(\boldsymbol{Z}_i^\top\boldsymbol{\beta})}{1-\exp\left\{-\sum_{L_i<t_k\le R_i}\lambda_k\exp(\boldsymbol{Z}_i^\top\boldsymbol{\beta})\right\}},\quad \text{if}\quad L_i<t_k\le R_i\quad\text{and}\quad R_i<\infty.$$

In the M-step, by setting $\partial Q(\boldsymbol{\theta})/\partial \lambda_k = 0$, we can update λ_k with the following closed-form expression

$$\lambda_k = \frac{\sum_{i=1}^n I(A_i \le t_k \le R_i^*) E(W_{ik})}{\sum_{i=1}^n I(A_i \le t_k \le R_i^*) \exp(\boldsymbol{Z}_i^T \boldsymbol{\beta})}, \ k = 1, \ldots, K_n.$$

By substituting the estimator of each λ_k above into $Q(\boldsymbol{\theta})$, we obtain the score equation for $\boldsymbol{\beta}$ as

$$\sum_{i=1}^n \left\{ \sum_{k=1}^{K_n} \left[I(A_i \le t_k \le R_i^*) E(W_{ik}) \left(\boldsymbol{Z}_i - \frac{\sum_{i=1}^n I(A_i \le t_k \le R_i^*) \exp(\boldsymbol{Z}_i^\top \boldsymbol{\beta}) \boldsymbol{Z}_i}{\sum_{i=1}^n I(A_i \le t_k \le R_i^*) \exp(\boldsymbol{Z}_i^\top \boldsymbol{\beta})} \right) \right] \right\} = 0.$$

Repeat the E-step and M-step until the convergence is achieved, and the covariance estimation of $\widehat{\boldsymbol{\beta}}$, the estimate of $\boldsymbol{\beta}$, can be accomplished by the profile likelihood approach given in Zeng et al. (2016) and others.

References

Zeng, Donglin and Mao, Lu and Lin, DY (2016). Maximum likelihood estimation for semiparametric transformation models with interval-censored data. *Biometrika.* 103(2), 253-271.
